# Parrondo’s Games Based on Complex Networks and the Paradoxical Effect

**DOI:** 10.1371/journal.pone.0067924

**Published:** 2013-07-02

**Authors:** Ye Ye, Lu Wang, Nenggang Xie

**Affiliations:** Department of Mechanical Engineering, Anhui University of Technology, Anhui, People’s Republic of China; Cinvestav-Merida, Mexico

## Abstract

Parrondo’s games were first constructed using a simple tossing scenario, which demonstrates the following paradoxical situation: in sequences of games, a winning expectation may be obtained by playing the games in a random order, although each game (game A or game B) in the sequence may result in losing when played individually. The available Parrondo’s games based on the spatial niche (the neighboring environment) are applied in the regular networks. The neighbors of each node are the same in the regular graphs, whereas they are different in the complex networks. Here, Parrondo’s model based on complex networks is proposed, and a structure of game B applied in arbitrary topologies is constructed. The results confirm that Parrondo’s paradox occurs. Moreover, the size of the region of the parameter space that elicits Parrondo’s paradox depends on the heterogeneity of the degree distributions of the networks. The higher heterogeneity yields a larger region of the parameter space where the strong paradox occurs. In addition, we use scale-free networks to show that the network size has no significant influence on the region of the parameter space where the strong or weak Parrondo’s paradox occurs. The region of the parameter space where the strong Parrondo’s paradox occurs reduces slightly when the average degree of the network increases.

## Introduction

Parrondo’s games can produce a paradoxical effect that alternating plays of two losing games can produce a winning game. Parrondo’s original games [1] involve two games, A and B. The player has some capital, which is increased by one with a probability of winning p_1_ and decreased by one with a probability 1-p_1_ in game A. Game B is slightly more complicated, and the rules are that if the capital is a multiple of an integer M, the probability of winning is p_2._ If it is not, the probability of winning is p_3_.

Game A is a losing strategy if p_1_<0.5. Harmer et al. [2] showed that game B is a losing strategy when the inequality 
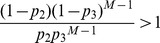
 holds, and the combined game A+B is a winning strategy when the inequality 
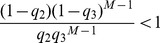
 holds, where 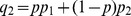, 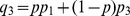, and p is the probability of playing game A. An example of the parameters that satisfy the previous inequalities was given by [1] as follows: p_1_ = 0.5-ε, p_2_ = 0.1-ε, p_3_ = 0.75-ε, M = 3, p = 0.5 and ε = 0.005. Notice that when ε is zero, game A is a fair game (i.e., it is neither losing nor winning on average).

Parrondo’s paradox has been confirmed by computer simulations, the Brownian ratchet and the discrete-time Markov chain [2]–[4]. In addition, the Parrondo effect has inspired the studies of the negative-mobility phenomena [5], the reliability theory [6], the noise-induced synchronization [7] and the controlling chaos [8]. In biological systems, Parrondo’s paradox may be related to the dynamics of gene transcription in GCN4 protein and the dynamics of transcription errors in DNA [2]. Moreover, Parrondo’s paradox has been studied in various interesting scenarios involving population genetics [9]–[12]. In the stock market, Boman et al. [13]–[14] used a Parrondian game framework as a toy model to study the dynamics of insider information. Parrondo’s paradox not only can be used to explain a large number of nonlinear phenomena [4] but also presents its own rich non-linear characteristics. Recent work [15] has shown that Parrondo’s games exhibit fractal patterns in their state space.

In Parrondo’s games, the construction of game B is a critical factor to produce the paradoxical phenomena. Usually, several asymmetric branches exist in game B, some of which are favorable (i.e., the probability of winning is large), and others are unfavorable (i.e., the probability of losing is large). These asymmetric structures form a “ratcheting” mechanism. When game B is played individually, game B is losing by setting the parameters of winning or losing probabilities. When two losing games are combined (game A+B) in a random or periodic alternation, the capital or the winning and losing states are changed because of the “agitating” role of game A. Thus, when it is game B’s turn to play, the chance for the favorable branches increases. Finally, a winning counter-intuitive phenomenon appears. This paradoxical effect has motivated a dynamic mechanism study of a widespread counter-intuitive phenomenon that exists in physics, biology, economics and other disciplines. In the engineering literature it is known that individually unstable systems can become stable if coupled together [16]. In the theory of granular flow, drift can occur in a counterintuitive direction. For instance, the “Brazil nut effect” [17]–[18] has shown that the large Brazil nuts rise to the top when you shake a bag of mixed nuts. In the area of mathematics, Pinsky and Scheutzow [19] have shown that switching between two transient diffusion processes in random media can form a positive recurrent process, which can be viewed as a continuous-time version of Parrondo’s games. Masuda and Konno [11], who studied the Domany-Kinzel probabilistic cellular automata, concluded that alternating two supercritical dynamics resulted in the subcritical dynamics in which the population died out. Almeida et al. [20] have proposed a case that “chaos+chaos = order”, where the periodic mixing of two chaotic dynamics resulted in an ordered dynamics under certain circumstances. Therefore, the dynamic mechanisms similar to Parrondo’s games exist behind the counterintuitive phenomenon produced by the two mixed systems (processes, states). One system plays the “ratcheting” role, and the other plays the “agitating” role. The key to study these counterintuitive phenomena is to analyze the “ratcheting” role. By analyzing the phenomena such as the Brownian ratchet, the Brazil nut effect, the longshore drift on a beach, the buy-low sell-high process in stock-market trading and the two-girlfriend paradox, Abbott [21] demonstrated that various “ratcheting” mechanisms were caused by the asymmetries of space, friction, information, money and time.

The structure of game B in Parrondo’s original games has two branches, which depend on the modulus of the capital. Such dependence limited the application of the games in practice. Therefore, Parrondo et al. [3] modified game B and devised a new structure that depended on the recent history (*t*-2, *t*-1) of wins and losses. The modified game B had four branches: (lose, lose), (lose, win), (win, lose) and (win, win). This new structure increased the region of the parameter space where the Parrondo’s paradox occurred. The theoretical analysis demonstrated that the paradoxical space based on the history-dependent model was 50 times larger than the original version [15].

The above variations of game B, which is capital-dependent or history-dependent, have been introduced into Parrondo’s games. Toral [22] and Mihailovic [23]–[24] proposed a structure of game B that depended on the neighboring players, who surrounded the player whose turn it was to play the game. A remarkable difference was that an ensemble of *N* players was considered instead of only one player. Each one occupied a certain space. For any player *i*, all of its surrounding neighbors composed its spatial niche. At present, there are networks of a one-dimensional line and a two-dimensional lattice, which connect *N* players. For the one-dimensional line, the neighbors of player *i* are *i*-1 and *i*+1. *I*–1 and *i*+1 have four different winning and losing states, which are (0 0), (0 1), (1 0) and (1 1), where 0 denotes a losing state, and 1 denotes a winning state. Therefore, the structure of game B consists of four branches. The probability of winning in each branch for player *i* is *p*_0_, *p*_1_, *p*_2_ and *p*_3_ respectively. For the two-dimensional lattice, the four neighbors of player *i* have the following five different winning and losing states (here, the positions of the winning and losing states of the neighbors are not distinguished): (1) all the four neighbors are in the losing states, that is, (0000); (2) one neighbor is in the winning state, and the other three neighbors are in the losing states, that is, (1000); (3) two neighbors are in the winning states, and the other two neighbors are in the losing states, that is, (1100); (4) three neighbors are in the winning states, and one neighbor is in the losing state, that is, (1110); (5) all the four neighbors are in the winning states, that is, (1111). Therefore, the structure of game B consists of five branches. The probability of winning in each branch for the player *i* is *p*_0_, *p*_1_, *p*_2_, *p*_3_ and *p*_4_ respectively.

Because the actual networks are complex, there are many types of topologies, such as random graphs, small-world networks and scale-free networks. However, the above two structures of game B cannot be extended directly to arbitrary topologies, where the number of the neighbors of the nodes cannot be maintained consistently. Therefore, we want to design a structure of game B that can be applied to arbitrary topologies, which means a structure of game B that can be applied to the nodes with different degrees. Toyota [25] proposed a construction method for game B in scale- free networks, where each player on a network played a game L when there was R or more winners in the neighborhood connected to the player and played a game W otherwise in game B. The simulation results and the theoretical studies showed that Parrondo’s paradox might not occur. Here, based on the spatial neighboring environment, the paper proposes a structure of game B to be applied in arbitrary topologies. The results show that Parrondo’s paradox can occur. Moreover, the size of the region of the parameter space that elicits Parrondo’s paradox depends on the heterogeneity of the degree distributions of the networks. A higher heterogeneity of the degree distributions of the networks produces a larger region of the parameter space where the strong paradox occurs.

## Model

**Figure 1:**
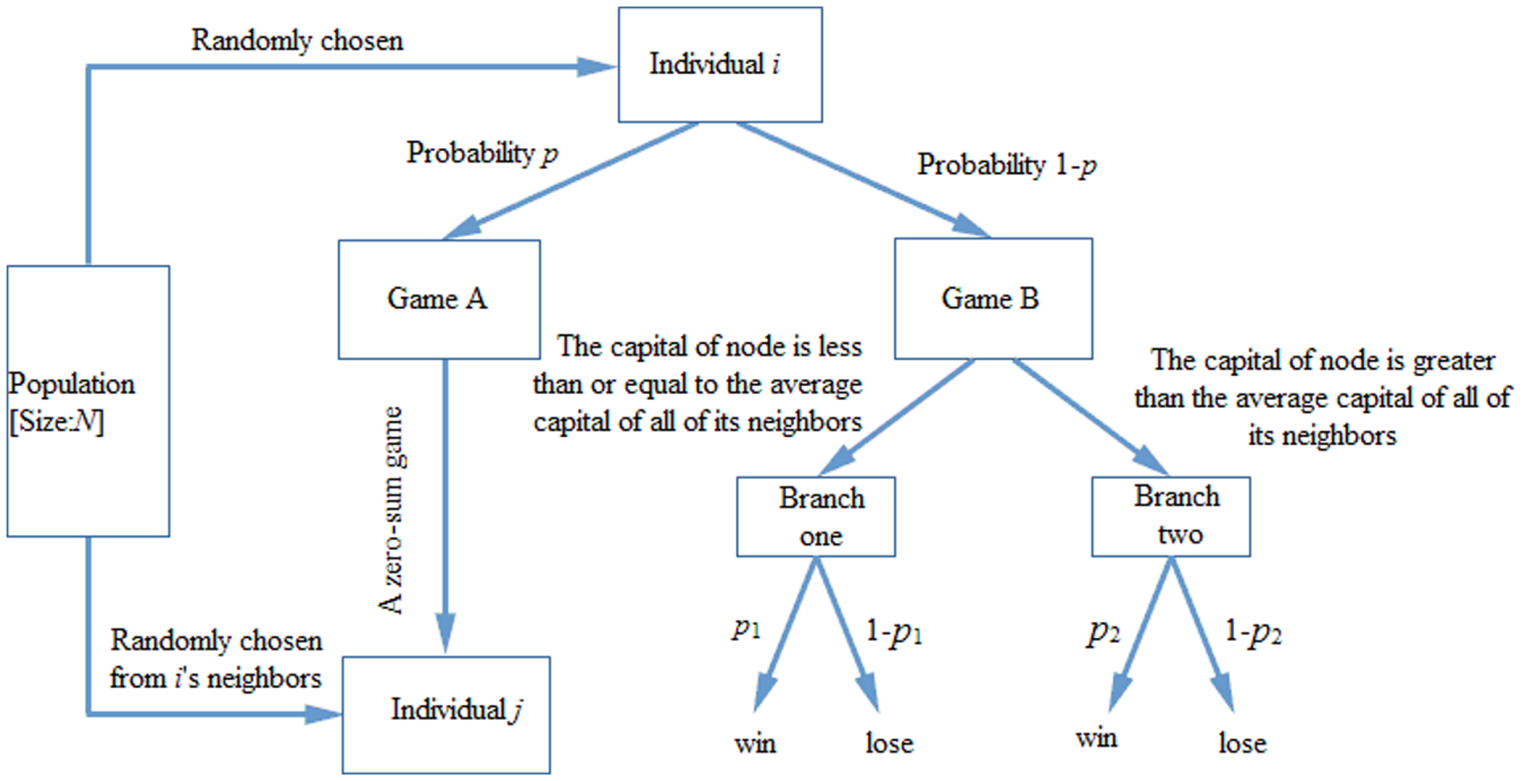
The structure of game B based on arbitrary topologies.

In this article, Parrondo’s model based on arbitrary topologies is shown in Figure 1. The model is composed of two games, A and B. Consider complex networks composed of *N* nodes. The game modes include playing game A and game B individually and playing a randomized game A+B. The randomized game A+B means a probabilistic sequence of games A and B. The dynamic processes of the randomized game A+B are as follows: for each round of the game, one node ‘*i*’ is chosen at random from *N* nodes to play game A (with a probability *p*) or game B (with a probability 1-*p*).

### A Zero-sum Game between Individuals–Game A

Game A is used to represent the competition behavior between the individuals in the networks, which is designed to be a zero-sum game. It has no impact on the total capital of the population, but it changes the capital distributions among the population. When game A is played, we need to randomly choose a node *j* from the neighboring nodes that are connected with *i*. The winning probabilities of nodes *i* and *j* are 0.5, respectively. When *i* wins, *j* pays one unit to *i*; conversely, *i* pays one unit to *j*.

### The Construction of Game B

We consider the following two conditions when constructing game B: (1) The structure of game B needs several branches to form the “ratcheting” mechanism. (2) The structure of game B needs to be applied to the individuals with an arbitrary number of neighbors. We must avoid the influence of the number of neighbors while constructing the branches. Based on these two considerations, game B has two branches, which are generated according to the capital of node *i* and its neighbors. In branch one, when the capital of node *i* is less than or equal to the average capital of all of its neighbors, the winning probability is *p*_1_; in branch two, when the capital of node *i* is greater than the average capital of all of its neighbors, the winning probability is *p*_2_. Game B can also be constructed in other forms that are applicable to any networks. Therefore, the results and the related conclusions of this paper are obtained based on the Parrondo’s model shown in Figure 1.

## Computer Simulations

We perform the following computer simulations based on Parrondo’s model in complex networks.

Based on the needs of computer simulations, we define the average fitness of the population *d* as follows:
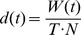
(1)where: *N* is the population size, and *T* is the average number of playing times for each individual. In this study, *T* = 100. 
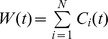
, which denotes the total capital of the population, and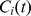 is the capital at time *t* (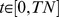) of individual *i*. The initial capital of all individuals is equal.

The condition under which the weak paradox occurs is as follows:
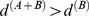
(2)


The conditions under which the strong paradox occurs are as follows:
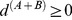
(3)

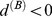
(4)where 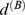
and 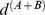
represent the capital of games B and A+B in the steady state, respectively.

In the following section, we analyze three types of factors, including the heterogeneity of the degree distributions of the networks, the network size and the average degree of the network, and observe the influence of these factors on the paradoxical effect.

### The Influence of the Heterogeneity of the Degree Distributions of the Networks on the Paradoxical Effect

To cleanly control the heterogeneity manipulation, we use the following two methods.

**Figure 2:**
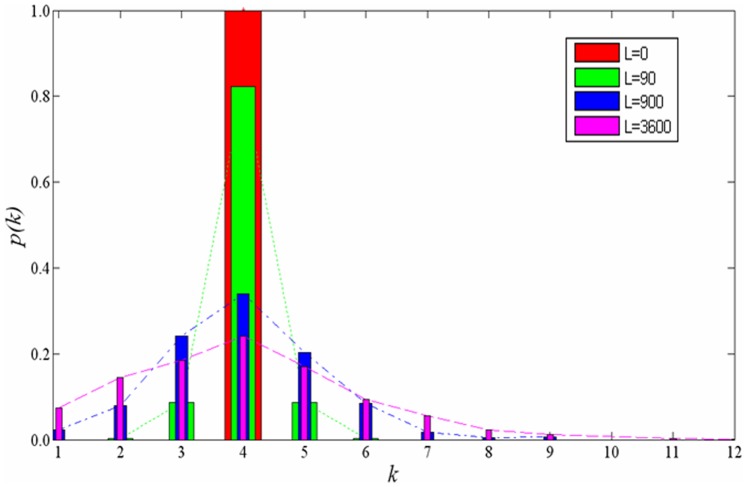
The degree distributions progressively changing from a two-dimensional network to a random graph. The size of the network is 900. The number of rewiring times *L* is 0, 90, 900 and 3600 respectively. *L* = 0 corresponds to a two-dimensional lattice. The degree distribution of the network is a δ distribution and the degree of all nodes is four. The node degree of the network progressively changes to a Poisson distribution with the increment of *L.* The average degree of the nodes remains at four during this process.

**Figure 3:**
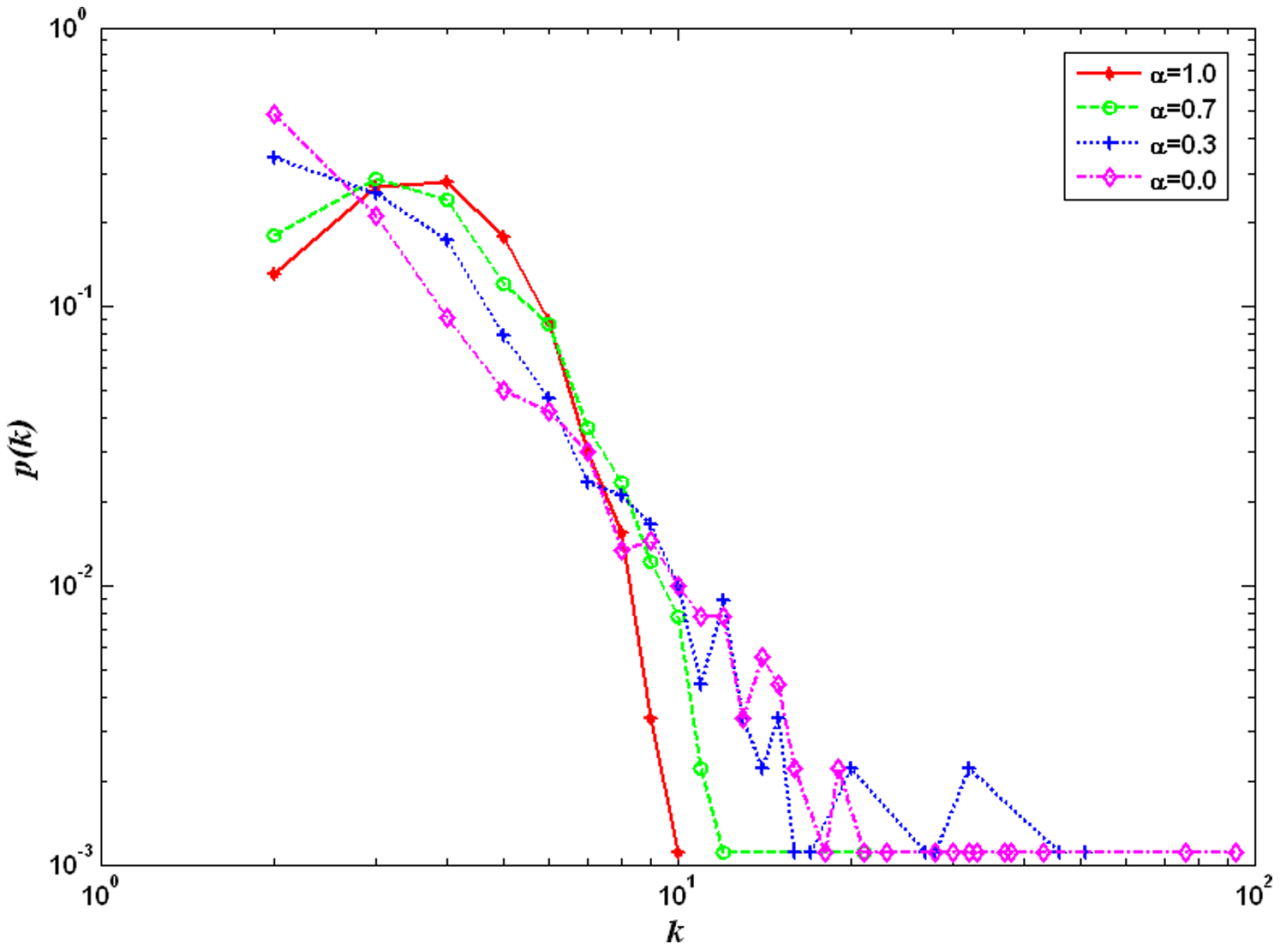
The degree distributions progressively changing from a random graph to a scale-free network. The network size is 900. The parameter α is 1.0, 0.7, 0.3 and 0.0 respectively. α = 1 corresponds to a random graph. The node degree of the network satisfies a Poisson distribution. With the decrease of α, the node degree of the network gradually changes to a power-law distribution. α = 0 corresponds to a scale-free network. The degree distribution of the network has an obvious fat-tail phenomenon.

1. In order to reflect the progressive changes from a two-dimensional lattice to a random graph, we start from a two-dimensional lattice and use the following rewiring mechanism to generate a random graph. The basic steps are as follows [26]: (a) generate an initial two-dimensional lattice; (b) randomly choose a node *E* and then randomly choose a node *F* from *E*’s neighbors. Break the connection between nodes *E* and *F*; (c) randomly choose two nodes *G* and *H* from the network and build a connection between *E* and *G* and a connection between *F* and *H*; (d) repeat steps (b) and (c) *L* times. As the number of rewiring times (*L*) increases, the stochastic degree of the network increases. The node degree of the network progressively changes from a δ distribution to a Poisson distribution. Moreover, the average degree of the network remains at four. The number of rewiring times *L* is the controlling index of the heterogeneity of the degree distributions. The corresponding degree distributions of the networks are shown in Figure 2 (where *k* is the degree, and *p*(*k*) is the probability).
2. To reflect the progressive changes from a random graph to a scale-free network, we use a model based on the degree distribution with adjustable parameters of the network and use the corresponding construction algorithm. The steps of the algorithm [27] are as follows: (a) growth. The initial network consists of *N* nodes, where *m*_0_ nodes are fully connected, and a set *J*_2_ is constituted; an unconnected set *J*_1_ is composed of (*N*-*m*_0_) isolated nodes. At each time step, choose a new node from *J*_1_. The new node has *m* edges connected with the other nodes. (b) Preferential attachment. The above new node’s *m* edges are randomly linked to any other node from (*N*-1) nodes with a probability α (here, we have to avoid the multiple edges); then connect the nodes of the set *J*_2_ by following a linear preferential-attachment strategy with the probability 1- α. After the connection is completed, we remove the new node from *J*_1_ and add it into *J*_2_. (c) After *N*-*m*_0_ time steps, a series of networks are generated by controlling the parameter 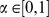, where α = 0.0 corresponds to a scale-free network, and α = 1.0 corresponds to a random graph. With a decrease in α, the node degree changes progressively from a Poisson distribution to a power-law distribution, and the average degree of the network remains at four. The parameter α is the controlling index of the heterogeneity of the degree distributions. Figure 3 shows the corresponding degree distributions of the networks.

**Figure 4:**
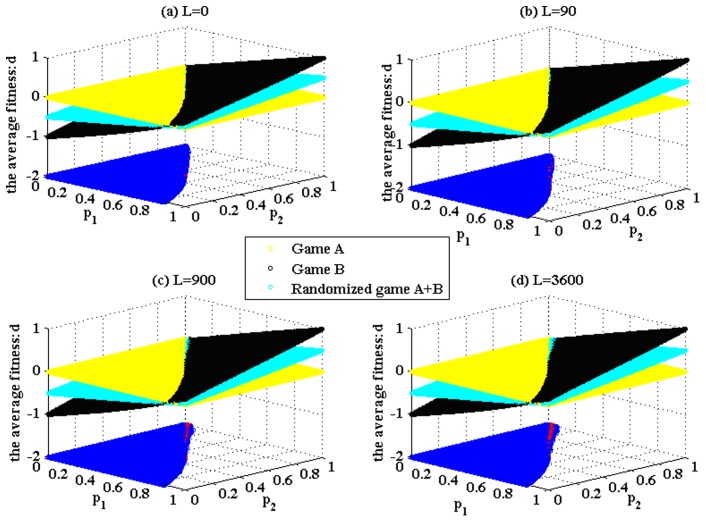
Simulations based on the networks, which progressively change from a two-dimensional lattice to a random network. The population size *N* is 900. The average degree of the network is four, and the average number of playing times *T* of each individual is 100. The probability of playing game A is *p* = 0.5. Play the games 30 times with different random numbers, and draw the corresponding figures according to the average results of the games. The blue area in the picture demonstrates the weak Parrondo’s paradox area, whereas the red denotes the strong area. Figures 4 (a), (b), (c) and (d) correspond to the networks with the rewiring times L = 0, 90, 900 and 3,600, respectively.

**Figure 5:**
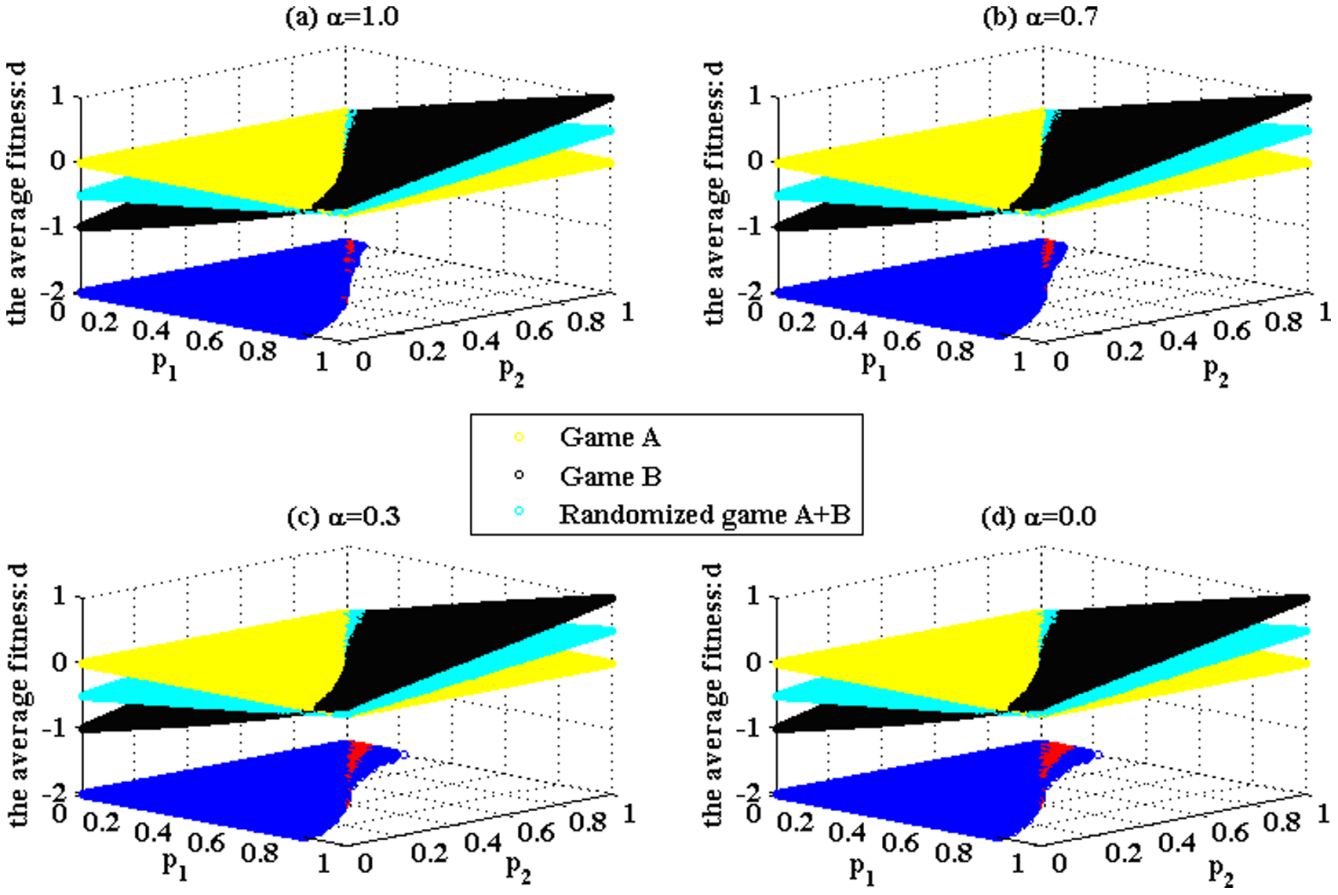
Simulations based on the networks, which progressively change from a random network to a scale-free network. The population size *N* is 900. The average degree of the network is four and the average number of playing times *T* of each individual is 100. The probability of playing game A is *p* = 0.5. Play the games 30 times with different random numbers, and draw the corresponding figures according to the average results of the games. The blue area in the picture demonstrates the weak Parrondo’s paradox area, whereas the red denotes the strong area. Figures. 5 (a), (b), (c) and (d) correspond to the networks with the parameter α = 1.0, 0.7, 0.3 and 0.0, respectively.

The simulation results based on Parrondo’s model (shown in Figure 1) are given in Figures 4 and 5. These results demonstrate that the structure of game B proposed in the paper can produce Parrondo’s paradox based on the complex networks. Figure 4 shows that the results of Parrondo’s paradox change progressively from a two-dimensional lattice to a random network. As the number of rewiring times, *L*, increases, the node degree of the network gradually changes from a 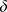
distribution to a Poisson distribution. Moreover, the region of the parameter space where the strong paradox occurs (the red region in Figure 4) grows progressively. The region of the parameter space where the weak paradox occurs (the blue part in Figure 4) is divided into two regions by the red part. The characteristic of the large blue area on the left of the red region is 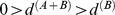. The characteristic of the small blue area on the right of the red region is 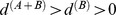. With the increment of the rewiring times *L*, the blue area corresponding to 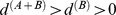 progressively grows. Figure 5 shows the results of Parrondo’s paradox progressively changing from a random network to a scale-free network. As the parameter α decreases, the node degree of the network progressively changes from a Poisson distribution to a power-law distribution. In addition, the region of the parameter space in which the strong paradox occurs (the red region in Figure 5) grows progressively. Moreover, the region of the parameter space in which the weak paradox occurs (the blue part that corresponds to 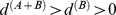, as shown in Figure 5) also grows. Therefore, the region of the parameter space where Parrondo’s paradox occurs is related to the heterogeneity of the degree distributions of the networks. A higher heterogeneity leads to a larger region of the parameter space where the strong or weak paradox occurs.

To analyze the mechanisms that elicit the paradox and the reason for the effect of the heterogeneity of the degree distributions of the networks, a set of specific parameters (*p*_1_ = 0.175 and *p*_2_ = 0.870) is chosen based on the results of Figures 4 and 5. We count the proportion of playing the favorable branch of game B, which is the number of times that the favorable branch of game B is played in comparison to the number of times that game B is played (for parameters *p*_1_ = 0.175 and *p*_2_ = 0.870, branch two is the favorable one). The results are shown in Tables 1 and 2.

**Table 1:** The average fitness and the proportion of playing branch two (from the two-dimensional lattice to the random network).

Game Modes	*L* = 0		*L* = 90		*L* = 900		*L* = 3600	
	*d*	proportion	*d*	proportion	*d*	proportion	*d*	proportion
Game B	0.0339	49.20%	0.0328	49.12%	0.0240	48.48%	0.0179	48.05%
Randomized game A+B	0.0187	49.47%	0.0186	49.44%	0.0172	49.25%	0.0166	49.15%

**Table 2:** The average fitness and the proportion of playing branch two (from the random network to the scale-free network).

Game Modes	α = 1.0		α = 0.7		α = 0.3		α = 0.0	
	*d*	proportion	*d*	proportion	*d*	proportion	*d*	proportion
Game B	0.0207	48.26%	0.0157	47.89%	−0.0092	46.10%	−0.0459	43.44%
Randomized game A+B	0.0172	49.23%	0.0163	49.11%	0.0155	48.99%	0.0138	48.75%

Tables 1 and 2 show that (1) the proportion of playing the favorable branch of the randomized game A+B is larger than that of game B played individually, which shows that the “agitating” role of game A increases the chance to play the favorable branch; (2) when the heterogeneity of the degree distributions of the networks increases, for the given set of parameters (*p*_1_ = 0.175 and *p*_2_ = 0.870), first, the paradoxical effect is not produced (when *L* = 0, 90, 900 and 3,600 and α = 1.0, 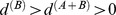), then, the weak paradox occurs (when α = 0.7, 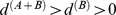), and finally, the strong paradox occurs (when α = 0.3 and α = 0.0, 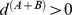 and 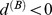). The same changing trend is shown in Figures 4 and 5. The main reason for this finding is that when the heterogeneity of the degree distributions of the networks increases, the proportions of playing the favorable branch in both the randomized game A+B and game B decrease. However, the proportion of playing the favorable branch in game B decreases more significantly, from 48.26% (when α = 1.0) to 43.44% (when α = 0.0), whereas the proportion of playing the favorable branch in the randomized game A+B decreases less from 49.23% (when α = 1.0) to 48.75% (when α = 0.0). This asynchronous decline shows that the “agitating” role of game A contributes to the increase of the opportunity to play the favorable branch. In addition, this contribution has a positive correlation with the heterogeneity of the degree distributions of the networks. Therefore, the asynchronous decline between the two results makes 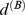
gradual changes from positive to negative, whereas 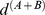 simultaneously remains positive. The given set of parameters with which the paradox originally does not occur gradually evolves into the set of parameters with which the weak paradox or the strong paradox occurs. (3) when *L* = 0, 90, 900 and 3,600 and α = 1.0, we find that each proportion of playing the favorable branch in game B is slightly smaller than that in the randomized game A+B, but 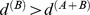. The reason for this result is that when we play the randomized game A+B, half of the total time is used to play the zero-sum game (i.e., game A). Although the proportion of playing the favorable branch in game B is smaller, the total time to play the favorable branch is more than that in the randomized game A+B (The total time of playing the unfavorable branch in game B is also more than that in the randomized game A+B). When the proportion of playing the favorable branch in game B is large enough, the corresponding gain is enough to offset the loss of playing the unfavorable branch. Therefore, 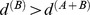.

In the following section, we use a BA network as an example and attempt to explain the following questions from a micro level: (1) why does a higher heterogeneity of the degree distributions of the networks lead to a smaller opportunity to play the favorable branch in game B? (2) Why does the “agitating” role of game A increase the chance to play the favorable branch? (3) The “agitating” role of game A contributes to increasing the opportunity to play the favorable branch. Then, why does this contribution have a positive correlation with the heterogeneity of the degree distributions of networks?

**Figure 6:**
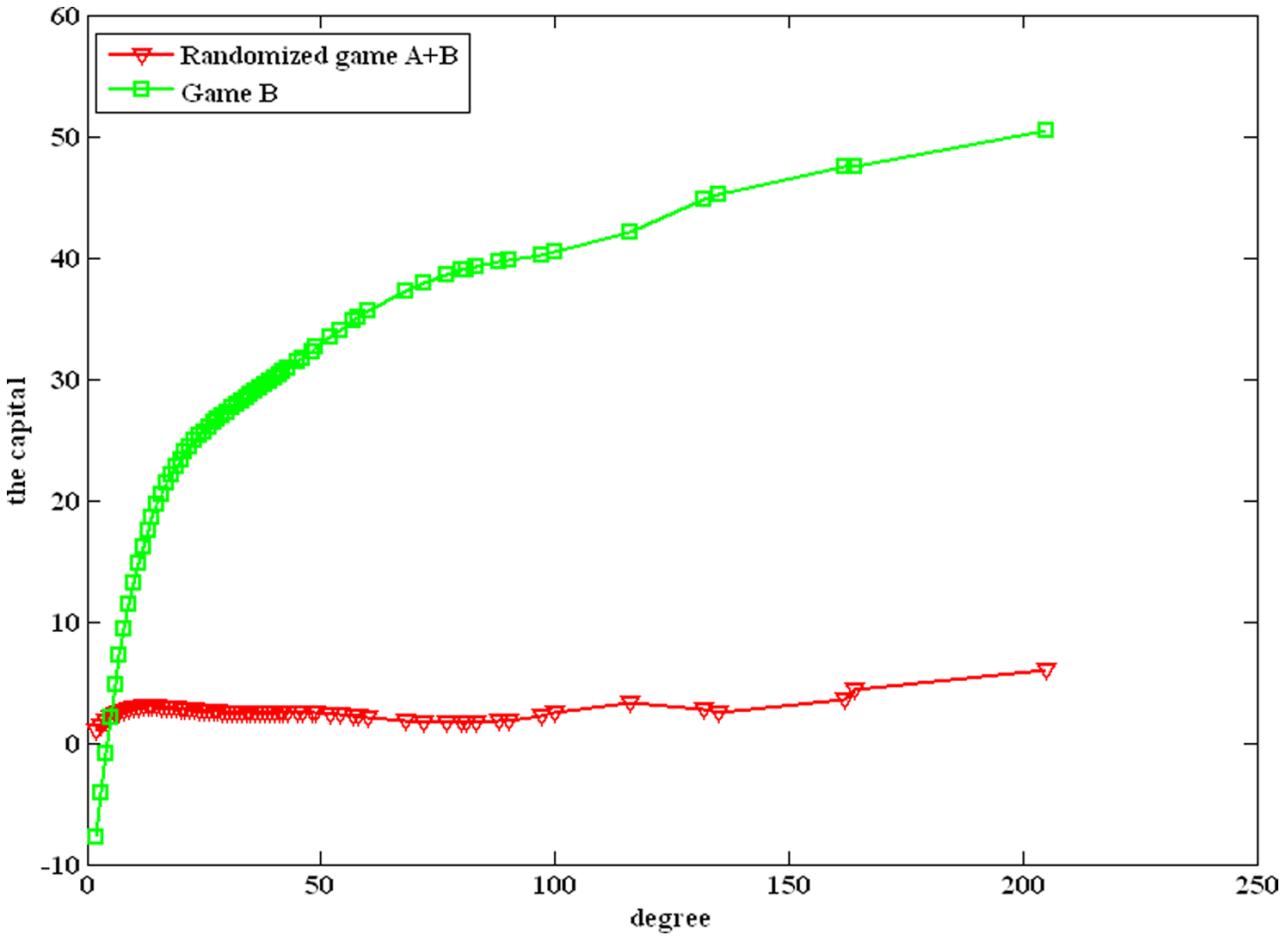
The relationship between the degree and the capital (based on the BA scale-free network). The population size *N* is 10, 000. The average degree of the network is four and the average number of playing times *T* of each individual is 100. The probability of playing game *A* is *p* = 0.5. The probabilities of winning in branch one and branch two of game B are *p*_1_ = 0.175 and *p*_2_ = 0.870, respectively. Play the games 1000 times with different random numbers, and draw the corresponding figures according to the average results of the games. For the nodes with the same degree, the capital is averaged from all of these nodes.

We choose *p*_1_ = 0.175 and *p*_2_ = 0.870 and perform the simulations on a BA network with 10,000 nodes. The results show that the average fitness values of the population *d* of game B and the randomized game A+B are −0.0456 and 0.0141, respectively. Thus, the strong paradox occurs. The proportions of playing the favorable branch in game B and the randomized game A+B are 43.48% and 48.79%, respectively. Figure 6 shows the relationship between the node degree and the capital. From the figure, we observe that when game B is played individually, the positive relations exist between the node degree and the capital. A larger node degree corresponds to a larger capital. The capital of node degrees two and three is negative and the capital of the other node degrees is positive (because the number of nodes with degrees two and three accounts for 70% of the total amount of nodes, the average fitness of the population is negative). This result occurs because for parameters *p*_1_ = 0.175 and *p*_2_ = 0.870, when the capital of a node is less than the average capital of all of its neighbors, the niche of this node is not favorable (branch one of game B is played, and the probability of winning is *p*_1_ = 0.175). Otherwise, if the average capital of all of the neighbors is less than or equal to the capital of a node, the niche of this node is favorable (branch two of game B is played and the probability of winning is *p*_2_ = 0.870). Because the niche of the nodes with large degrees is mainly composed of nodes with small degrees, we assume that the capital of the nodes with small degrees is small. At the beginning of the game, because the number of the node degrees two and three accounts for 70% of all nodes, the nodes with small degrees have a large chance of being chosen for the game. In addition, because the initial capital of all nodes is the same, according to the rules of game B, the node with a small degree chosen for the game will play branch one. Then, the probability of losing is large, which results in the capital decreasing. Therefore, in the initial stage of the game, this hypothetical situation is a large-probability event. Thus, the nodes with large degrees play the favorable branch two with a larger probability, which increases the capital of the nodes with large degrees. Therefore, the niche of the small-degree nodes that are connected to these large-degree nodes is further worsened (because the number of neighbors of the small-degree nodes is only two or three, the increment of the capital of the large-degree nodes causes the average capital of the niche to produce a comparatively obvious rise). Moreover, this result makes the small-degree nodes play the unfavorable branch one with a larger probability, which reduces the capital of the nodes with small degrees. The favorable niche of the nodes with large degrees and the unfavorable niche of the nodes with small degrees are constantly strengthened in the playing courses. Finally, a phenomenon is produced that the larger node degree corresponds to more capital, and the smaller node degree corresponds to less capital. Meanwhile, because the number of nodes with small degrees is far greater than the number of nodes with large degrees, the favorable niche of the nodes with large degrees and the unfavorable niche of the nodes with small degrees make the proportion of playing the favorable branch small. This situation, which is favorable for the nodes with large degrees and unfavorable for the nodes with small degrees, is quite obvious in the BA network. When the heterogeneity of the degree distributions of the networks decreases, this situation will be weakened. The proportion of playing the favorable branch of the population will increase.

When the randomized game A+B is played, from Figure 6, we can observe that the node degree has no obvious relation with the capital, where the capital of the node degrees two and three, which account for 70% of the population, is positive (this result makes the average fitness of the population is positive). The reason for this result is the “agitating” role of game A. Because the nodes with large degrees and the nodes with small degrees will play a zero-sum game among them, the winning and the losing probabilities are the same. This process makes the nodes with small degrees have the chance of capital growth. Moreover, this process disrupts the strengthening process of the favorable niche of the nodes with large degrees and the unfavorable niche of nodes with small degrees. Even in a local area of the network, an inverse strengthening process may appear, where the unfavorable niche of the nodes with large or medium degrees (the average capital of the neighbors of the nodes with small degrees is large) and the favorable niche of the nodes with small degrees are formed (in this example, there is a phenomenon that the capital of nodes with degrees 49, 81, 83 and 97 is negative). Therefore, the “agitating” role of game A makes the nodes with small degrees increase the chance of playing the favorable branch in game B.

A higher heterogeneity of the degree distributions corresponds to a larger proportion of pairs of neighbors that are composed of large-degree nodes and small-degree nodes. Moreover, a higher heterogeneity of the degree distributions correspons to a larger probability that the nodes with large degrees and the nodes with small degrees play game A. Thus, this process disrupts the strengthening process that the favorable niche is formed by the nodes with large degrees and the unfavorable niche by the nodes with small degrees. Therefore, the “agitating” role of game A contributes to the increas of the opportunity to play the favorable branch, and this contribution positively correlates with the heterogeneity of the degree distributions of the networks.

### The Influence of the Network Size on the Paradoxical Effect

**Figure 7:**
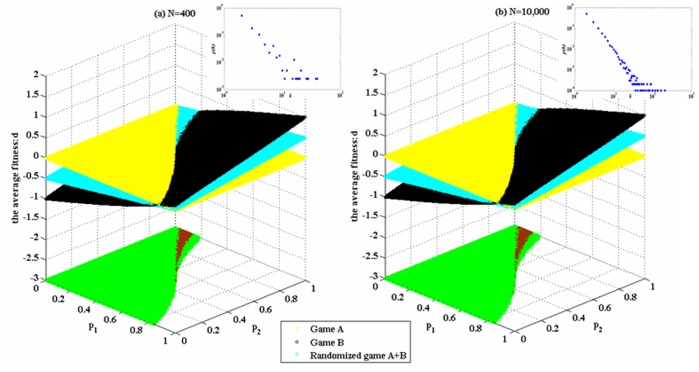
The influence of the network size on the Parrondo effect (based on the BA scale-free network). The average degree of the network is four and the average number of playing times *T* of each individual is 100. The probability of playing game A is *p* = 0.5. Play the games 30 times with different random numbers, and draw the corresponding figures according to the average results of the games. The small window in the figure shows the degree distribution of the nodes. The green area in the figure represents the area of the weak Parrondo’s paradox, whereas the brown region denotes the strong area. The population size *N* of Figure 7 (a) is 400 and Figure 7 (b) is 10, 000, respectively.

In order to investigate the impact of the size of the scale-free network on the Parrondo effect, we maintain the same average degree of the network (i.e., four) and the same heterogeneity of the degree distributions of networks. The size of the scale-free network is reduced to *N* = 400 and expanded to *N* = 10,000, respectively. The results are shown in Figure 7. Comparing Figure 5 (d) with Figure 7 (a) and Figure 7 (b), we notice that the region of the parameter space where the strong or weak paradox occurs has no significant change with the expansion of the network size (in the region of the parameter space where the strong paradox occurs, the percentages of Figure 5(d), Figure 7(a) and Figure 7(b) are 1.71%, 1.74% and 1.72, respectively; in the region of the parameter space where the weak paradox occurs, the percentages of Figure 5(d), Figure 7(a) and Figure 7(b) are 50.38%, 50.46% and 50.53%, respectively). The reason for this result is that when the average degree of nodes remains unchanged, the expansion of the size of the scale-free network has not effectively changed the heterogeneity of the degree distributions of networks.

### The Influence of the Average Degree of Networks on the Paradoxical Effect

**Figure 8:**
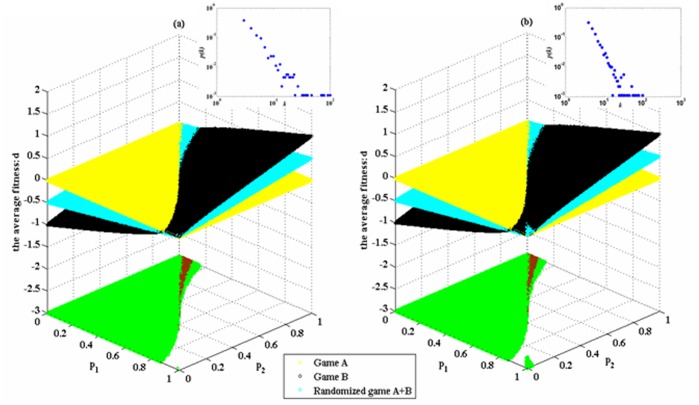
The influence of the average degree of the nodes on the Parrondo effect (based on the BA scale-free network). The population size *N* is 900 and the average number of playing times *T* of each individual is 100. The probability of playing game A is *p* = 0.5. Repeatedly play the games 30 times with different random numbers, and draw the corresponding figures according to the average results of the games. The small window in the figure shows the degree distribution of the nodes. The green area in the figure represents the weak-paradox area, whereas the brown region denotes the strong area. The average degree of the network in Figure 8 (a) and Figure 8 (b) is 5.9867 and 7.9756, respectively.

In addition, in order to investigate the impact of the average degree of the scale-free network on the Parrondo effect, we maintain the same network size (i.e., *N* = 900) and the same heterogeneity of the degree distributions of networks. We increase the average degree of the scale-free network. The results are shown in Figure 8. We compare Figure 5 (d) (the average degree of the network is four) with Figure 8 (a) (the average degree of the network is 5.9867) and Figure 8 (b) (the average degree of the network is 7.9756). Then we notice that the region of the parameter space where the strong Parrondo’s paradox occurs slightly reduces (the percentages of Figure 5 (d), Figure 8(a) and Figure 8(b) are 1.71%, 1.37% and 1.17%, respectively) when the average degree of the network increases. When the size of the scale-free network remains unchanged, the increment of the average degree of the node has slightly reduced the heterogeneity of the degree distributions of networks. The region of the parameter space where the weak paradox occurs has no significant change (the percentages of Figure 5 (d), Figures 8 (a) and 8(b) are 50.38%, 50.37% and 50.40%, respectively). In addition, comparing Figure 5(d) with Figure 8(a) and Figure 8 (b) between the regions of p_1_→1 and p_2_→0, we notice that the region of the parameter space where the weak Parrondo’s paradox occurs increases with a small growth (the percentages of Figure 5 (d), Figure 8 (a) and Figure 8 (b) are 0%, 0.005% and 0.25%, respectively) along with the increment of the average degree of the network. The capital corresponding to these areas is 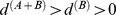.

### The Influence of the Probability *p* of Playing Game A on the Paradoxical Effect

**Figure 9:**
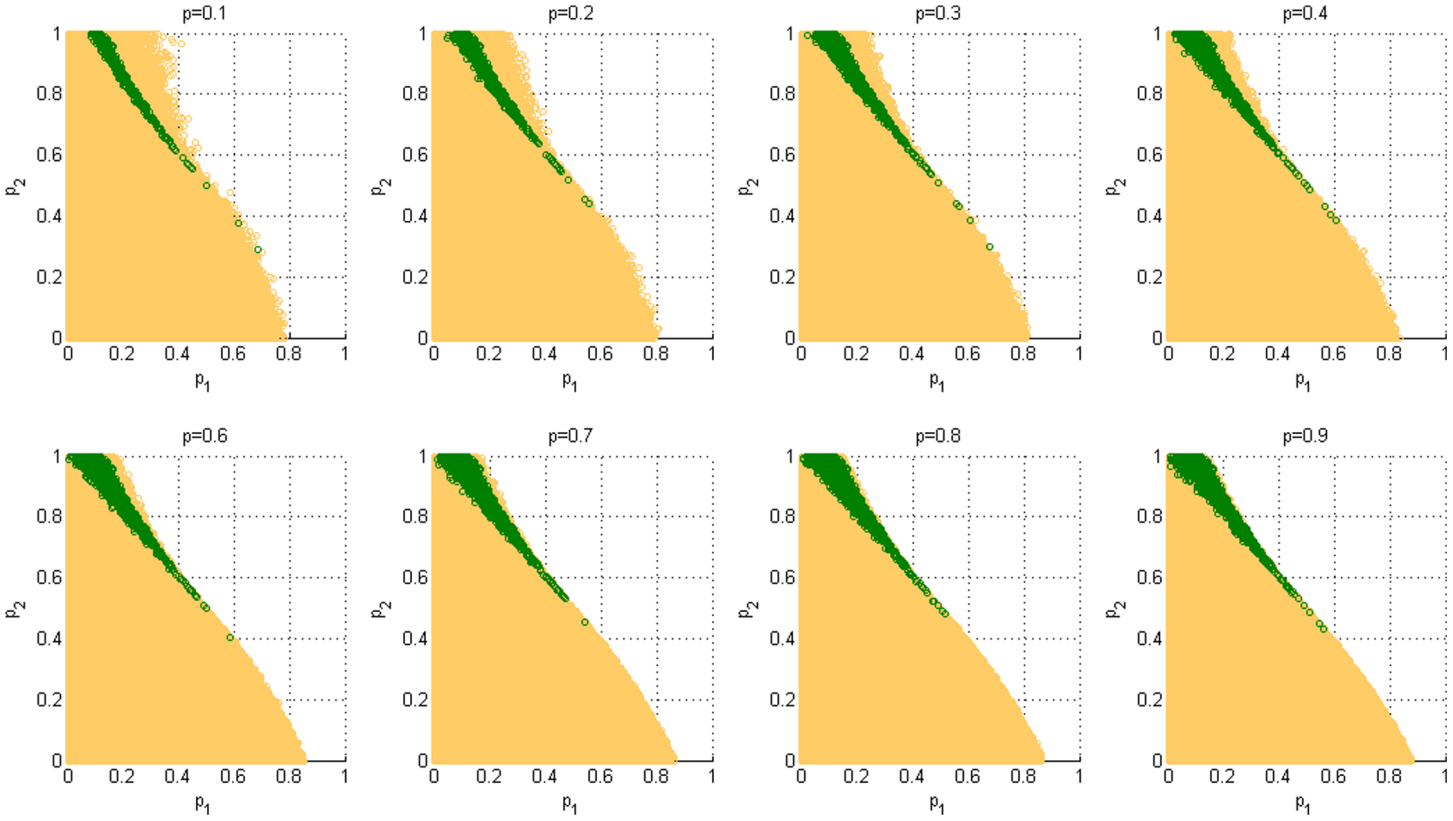
The influence of the probability *p* of playing game A on the Parrondo effect (based on the BA scale-free network). The population size *N* is 900. The average degree of the network is four and the average number of playing times *T* of each individual is 100. Play the games 30 times with different random numbers, and draw the corresponding figures according to the average results of the games. The orange area in the figure represents the weak-paradox area, whereas the green region denotes the strong-paradox area.

Finally, in order to reflect the effect of the probability *p* of playing game A, based on a scale-free network, we calculate the capital of the strong and weak regions that corresponds to different *p* values, as shown in Figure 9. From Figures 9 and 5(d), we notice that when *p*≤0.5, the strong paradoxical region gradually increases with the increment of *p*. The weak paradoxical region of the upper part of the trapezoidal shape (which corresponds to 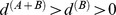) gradually reduces with the increment of *p*, whereas the weak paradox region of the bottom part of the trapezoidal shape (which corresponds to 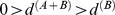) gradually increases with the increment of *p*. When *p*>0.5, the strong paradoxical region has no obvious change with the increment of *p*. With the increment of *p*, the weak paradoxical region that corresponds to 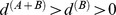 reduces gradually, whereas the weak paradoxical region that corresponds to 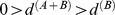 has no obvious change. Therefore, playing game A with a larger probability (*p*≥0.5) is conducive to generating a strong paradox.

## Conclusions

In this article, Parrondo’s model based on complex networks (shown in Figure 1) is proposed. Based on the spatial niche, a structure of game B is constructed, which can be applied to arbitrary topologies. The results show that Parrondo’s paradox occurs. Moreover, the region of the parameter space where Parrondo’s paradox occurs is related to the heterogeneity of the degree distributions of networks. A higher heterogeneity corresponds to a larger region of the parameter space where the strong paradox occurs. Therefore, the heterogeneity of the degree distributions of networks is conducive to the “agitating” mechanism. This mechanism may also cause most of the networks in reality to exhibit the high heterogeneity. In addition, the region of the parameter space where the strong or weak paradox occurs does not change significantly when the scale-free network expands. The region of the parameter space where the strong Parrondo’s paradox occurs slightly reduces when the average degree of the network increases. Moreover, playing game A with a larger probability is conducive to generating a strong paradox.
